# Challenges Regarding Water Quality of Eutrophic Reservoirs in Urban Landscapes: A Mapping Literature Review

**DOI:** 10.3390/ijerph16010040

**Published:** 2018-12-24

**Authors:** Sofia Oliver, Jason Corburn, Helena Ribeiro

**Affiliations:** 1 School of Public Health, University of São Paulo, São Paulo, SP 01246-904, Brazil; 2 College of Environmental Design, University of California, Berkeley, CA 94720-1870, USA; jcorburn@berkeley.edu

**Keywords:** water supply, urban health, environmental health, cyanobacteria, review literature, fresh water

## Abstract

Urbanized river basins usually suffer from anthropogenic pressure, compromising the quality of water. Unsafe water is a risk to public health, especially when there are occurrences of HABs (Harmful Algae Blooms) as in the case of cyanobacteria, which cause different human health problems. In this paper, we aimed to review the scientific literature documenting what has been studied in the scope of the stratified reservoirs of urbanized basins. The mapping review method was used to categorize existing literature on urbanized watersheds and eutrophic reservoirs. Using the keywords “Eutrophic Reservoir” and “Urban” and selecting all the years of open publication on the Science web page, we obtained 69 results, 53 of them meeting the requirements established for the search. Many of the studies mention as the most important determinant for eutrophication of reservoirs and the proliferation of algae, the anthropogenic influence through the diffuse load of streets, domestic and industrial sewage, and even drainage water from agricultural areas in the basin. The results of this study reinforce that informal settlements without sanitary infrastructure are aggravating the deterioration of water quality in urban water sources and therefore posing many risks to public health.

## 1. Introduction

Scarcity of water may be absolute lack of water, or may be due to inadequate exploitation and distribution, lack of access by part of population, and, in the case of drinking water, it can be related to its insufficient quality [1]. In this manuscript, we draw attention to deterioration of watersheds that may cause impediments to potable water access [1]. This is an issue from arid and distant backlands to humid environments where human life and technology abound: the cities.

Pressure of uncontrolled urban growth has been compromising existing water sources around the world. In low and middle income countries, the paucity of basic sanitation results in domestic sewage dumped into water bodies [2]. In addition, the shortage of adequate drainage and the increase of impervious soils by constructions and asphalt prevent soil recharge by the natural cycle. As consequence, contaminated runoffs, floods and torrents contribute to water quality loss [2].

As the city grows, the riparian vegetation might be substituted by improvised dwellings, compromising the health of the ecosystem and of the population living on the margins of reservoirs. The poorest and most vulnerable families often occupy those areas designated by law for preservation, because they are not part of the formal real estate market. There are also frequent irregular occupations of the margins by commercial ventures, clubs and high-level residences that enjoy the scenic advantages that the reservoirs offer.

The environmental impact of urban pressure on water sources is also compounded by contaminated untreated clandestine industrial effluents and by atmospheric pollution from industries and motorized transport carried by precipitation [2], contributing to the eutrophication of aquatic ecosystems [2].

Limnological imbalance caused by pollution as nutrient´s excess, triggers a succession of hindrances to drinking water´s potability, even after treatment. The consequence is cyanobacteria blooms, toxic algae that benefit from the abundance of organic matter and warm water temperatures [1]. Conventional treatment methods do not remove those toxic algae that are harmful to animals and humans [3].

Cyanobacteria are widely known for their potential to produce cyanotoxins. Depending on the type of toxin, such as microcystin, nodularins and cylindrospermopsins (hepatotoxins) [4] which are the most common, a slower poisoning may occur and can cause death of animals within a few hours or days, due to liver hemorrhage and hypovolemic shock [4].

Cyanotoxins can be hepatotoxic, neurotoxic, cytotoxic, dermatotoxic, carcinogenic and gastric irritants [2,3,4]. The effects on human health can range from liver and gastrointestinal disorders, neuromuscular dysfunction, allergic reactions, cancer and death [2,3,4].

Chronic exposure to cyanotoxins is potentially alarming given the scale of exposure of urban populations and their accumulative effect [2]. The blooms confer different color, odor and flavor to the water, causing problems for public supply of potable water and for the recreational use as well [4].

Growing world urbanization and climate change can accentuate the proliferation of these algae and the contamination of water sources [1,2], transforming it in a global phenomenon. This way, eutrophic urban reservoirs constitute public and environmental health problems, given the risk they pose to population´s health [5]. In this paper, we aimed to review the scientific literature documenting what has been studied in the scope of the stratified reservoirs of urbanized basins, focusing on cyanobacteria blooms as an environmental and public health issue.

## 2. Materials and Methods

A mapping literature review was undertaken [6] in order to map out and categorize existing studies on urbanized eutrophic watersheds, with articles found at the Web of Science Platform. We have chosen this platform because it gives access to multiple databases that reference cross-disciplinary research, which allows for in-depth exploration of specialized sub-fields within an academic or scientific discipline. It allowed a wide picture of what is being done on the topic.

We first looked for the words “Eutrophic reservoirs” resulting in 1586 studies, so we refined for “Eutrophic reservoirs” and “Cyanobacteria”, in order to know which of these contemplated the algae. This resulted in 357 studies, without limits of date or area. Among the indexed studies found in this initial phase, Brazil ranked as the first in numbers, with 72 publications; China followed with 38 publications; USA with 35; Poland with 26; and France with 23 publications. We found less than 5 manuscripts per country from Ethiopia, with 3 publications; Morocco, Nigeria, South Africa, Tunisia, Uganda, Vietnam and Singapore with 2 publications each.

From this starting point, we refined the search by adding the word urban, in order to know which of these studies were located in urbanized basins. We then chose to map out the eutrophic reservoirs found in urban basins using those results to explore further information. The aim was to collect the studies that mentioned eutrophic reservoirs in urban environments, because the eutrophic water body is already a threat for water quality, even if the study doesn´t mention HABs or Cyanobacteria, once the environment is ready for its frequent proliferation because of the water body´s trophic state and of rising temperatures.

This final search was done in November 2017, using the keywords “Eutrophic reservoirs” and “Urban”, setting no time span.

From 69 articles found, 16 were discarded because they did not fulfill the study requirements: the study had to refer both to eutrophic reservoirs and to urban settings. Therefore 53 articles have been selected and then systematized and mapped.

## 3. Results

**Figure 1:**
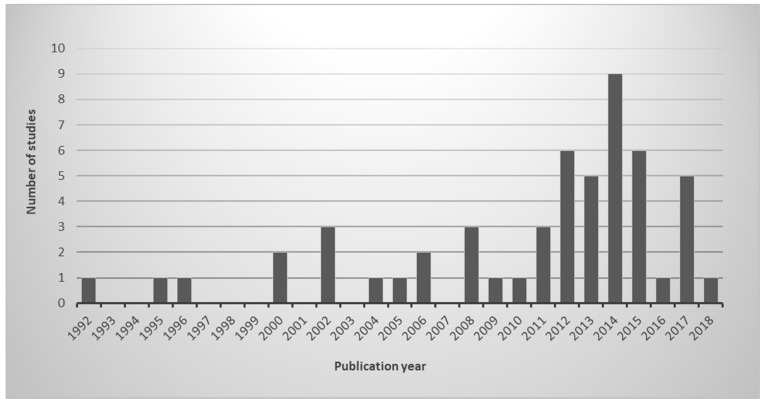
Number of studies on eutrophic urban reservoirs published per year.

As shown in Figure 1, there has been a growing interest in the problem of eutrophication of urban reservoirs in the last decade. This interest certainly shows an increase in the problem. Since the search was done in November 2017, results for the year 2018 do not appear. Brazil passed through an acute water crisis during the period 2014–2016 and those years experienced large algae blooms. Since most of the studies were undertaken in Brazil, it might explain the higher number of studies in 2014, 2015 and 2017, following that crisis.

Of the total, 32 of the studies were done to monitor the water quality of reservoirs, 11 studies aimed at the dynamics and peculiarities of living organisms in the limnological/aquatic environment. Ten (10) of the studies reported cases and techniques for reservoir recovery. Thus, this categorization was made to analyze the reviewed articles (Table 1).

Results exhibited in Table 1 with fields “Cy” (Cyanobacteria), “OA” (Other Algae) and “S/C” (Seasonality or Climate) refer to whether there was direct mention in the study of these terms and are indicated by “y” of yes, which means that the phenomenon was present and by “n” of “no” if the term was not contemplated. The “Country” field refers to the country in which the studied reservoir is located, regardless of the affiliation of the study researchers.

**Figure 2:**
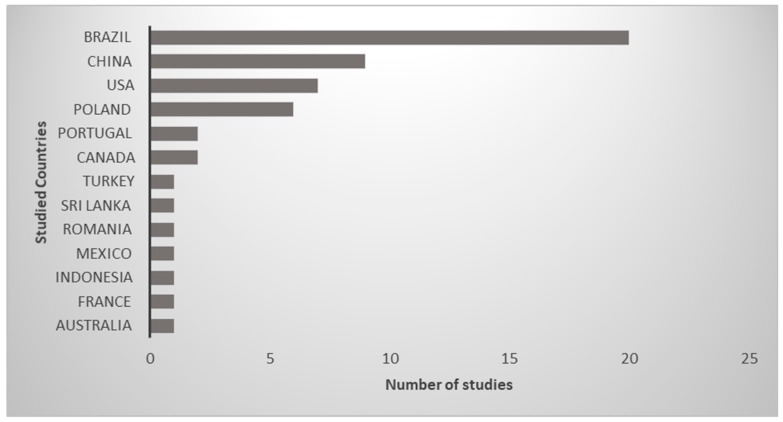
Countries where the eutrophic urban reservoirs were located 1992–2018.

Of the 53 studies selected in this literature mapping [6], 20 were made in Brazil, 9 in China, 7 in the United States and 6 in Poland, while Portugal and Canada hosted two studies each and the other countries only 1 each study, as can be seen in Figure 2.

In the 53 manuscripts, 39 reservoirs were studied as some merited more than one article, for they were analyzed in different aspects. The world spatial distribution of the studied reservoirs revealed locations in latitudes that span past the tropical and temperate zones, reaching cooler territories in the northern hemisphere. It was not possible to establish a distribution pattern of the studies according to the climatic classification of each area. This may indicate that aspects related to land use and water pollution play a more important role than climatic factors. However, the seasonality of time was presented in 28 of the studies (53%), indicating that variations in temperature, radiation, and precipitation are relevant factors for the proliferation of algae. The researches indicated as contributors to blooms: increased air temperatures [8], water temperature [21], stability of water column [7] reflecting weak winds, light penetration [44]. Regarding season of the year favorable for the proliferation, some studies refer to autumn [19,32,45], other mid summer [49], or refer to dry season [12] or to rainy season [44]. Thus, there is not a definite pattern regarding climate variables around the world. On the other hand, some studies reinforce that those undergoing sedimentation processes, and shallow rivers and reservoirs are more prone to eutrofization [15]. Major run offs [32,44] in rainy season are largely responsible for the process.

**Figure 3:**
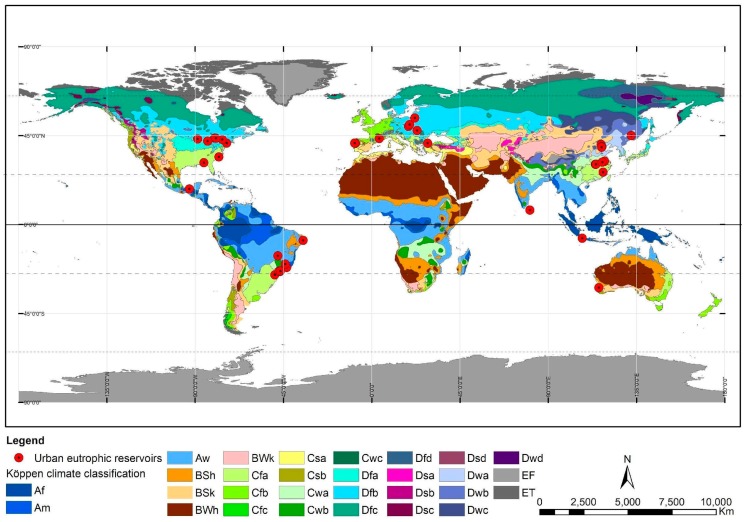
Spatial distribution of studies and climatic classification of Koppen-Geiger.

More than 50% of the studies are in zones of continental humid climate or continental climates, characterized by rains in the summer or throughout the year. The climatic classification of Köppen-Geiger is represented by the abbreviations Dfa, Dfb, Dwa and Dwb as in Figure 3. Other reservoirs, although smaller in number, are located in warmer climates that are rainy with dry winter, in the climatic zone Aw.

However, only one of them mentioned “climate change” as important in the investigation approach [7]. It dates back to 2017, one of the most recent of the bibliographical research, indicating that this theme has only recently been receiving interest in the field of limnology.

Investigations highlight the contribution of phosphorus [35] and nitrogen [21,24] in the water for the outbreak of algae blooms. Of the total manuscripts analyzed, regardless of the category in which they were classified, 25 described algal blooms in general; 20 mentioned cyanobacteria blooms; and 15 reported the occurrence of several species of algae, including cyanobacteria. Thus, the presence of algae represents a constant problem of urban reservoirs and, as mentioned, it may pose a serious threat to public health since many of these reservoirs are, or have the potential to be, used for water supply due to the proximity of populations. However none of the articles presented the health risks this poses, although 2 of them [24,36] mention “drinking water”, showing concern about quality for human consumption. Other articles show indirect concern for “environmental health” [9,22], “sustainable water resources” [22] or “propagation of antibiotic resistance genes in urban lakes” [9].

Even though the origin of the reservoirs was not the main object of the research, it allowed verifying its variability. Studies in Poland indicate that most of the reservoirs originated from mining caves and many were in the process of remediation. In China and Brazil, the reservoirs are mostly dammed rivers and streams. In temperate countries, reservoirs could be formed from snowmelt.

Among the 10 studies describing techniques used for reservoir remediation, 9 showed positive results and a decrease in the presence of toxic algae.

## 4. Discussion

The results of this research, done without time or space limits, indicated reduced numbers of investigated reservoirs considering that this is such an important topic in a context of great worldwide concern with water resources. It is a limited number of papers that consider the approach of the theme identifying the reservoir as urban, recognizing this environmental characteristic as an important factor. Another limitation of the research may be the fact that the database used searches only in indexed journals that have digitized collections.

Although literature indicates studies on these algae since 1992, the pioneering study was digitized in 2006. Other studies on the subject may have been done previously, although they have not been indexed in a bibliographic database. Our literature review shows that concerns and studies are growing in time. This may reflect an increase in the problem of reservoir eutrophication, which has been concentrated in large, less recent industrial urban centers, such as in the Great Lakes region of North America, the site of a significant number of studies on the subject. Nowadays, with the acceleration of urbanization in low- and middle-income countries, this problem reaches urban centers in these nations more intensely, mainly due to the deficits in sanitation they present, thus prompting further research. This is the case of Brazil, which has seen some of its water supply reservoirs, such as the Guarapiranga dam, threatened by the proliferation of cyanobacteria [5].

Many of the studies mention as the most important determinant for eutrophication of reservoirs and the proliferation of algae the anthropogenic influence through diffuse loads of streets, domestic and industrial sewage, and even runoffs from agricultural areas in the drainage basin. The sediments in the reservoirs and the nutrient loads of phosphorus and nitrogen are directly related to the soil occupation for urban settlement, and to the removal of natural vegetation [22,31]. However, those are not the only drivers: high values of coliforms due to untreated sewage, low values for diversity of epibenthic macrofauna and fish [23], air dried organic matter in shallow lake sediment [27], excretion by larvae at high temperatures area also important contributors, according to the studies cited in this review. Thus, there are many challenges regarding the control of eutrophication in cities reservoirs, streams and rivers.

The outbreak of cyanobacteria changes the functional phytoplankton group and zooplankton communities in urban eutrophic lakes [33,40], which are further altered by copper sulphate treatment [41] that might also alter the morphology of fish gills [42].

Even though the restoration studies, mentioned in this review, report good results in the control of nutrient load by wastewater treatment and prevention of diffuse sources to enter the water bodies, sewerage and adequate controls need also to be placed in regions upstream [21,35] and a selection of activities must be made by city government and residents. However, high costs, complexity of actions, and the long term seem to discourage decision makers.

The relationship found in some papers between climate variables and cyanobacteria blooms has raised concern that global climate change, by reinforcing favorable flowering time periods, could aggravate the frequency of the phenomenon [5], turning climate control another major challenge.

These studies cited should base programs and actions that aim to limit water pollution, and reinforce arguments for the relevance of facing this problem urgently, often by giving economic value to reservoirs, but above all to protect human and animal life. Reducing water pollution can be done by government policies such as zoning or land use regulations, even though extensive basic sanitation programs in cities of middle and low-income countries may chiefly achieve this reduction. The conservation of water sources can be an important strategy to safeguard water supplies for coming decades.

## 5. Conclusions

The quality of human water supply is, at the same time, a very old and a very recent topic. It is an area of interdisciplinary and multidisciplinary research and action that is gaining importance given the water scarcity that has affected populations in various parts of the globe, and because of large-scale climatic oscillations prompted increased attention to the issue. The proliferation of cyanobacteria, as well as the contamination of urban reservoirs, once considered topics of limnology, must also be considered a problem of urban planning and governance.

The number of articles found in this mapping literature search does not reflect the real environmental issue of eutrophic reservoirs in urban areas of the world. However, there might be more abundant studies on the topic in specific scientific areas exclusively. The importance of our study was to highlight the relation between urban land and eutrophication of reservoirs, and the risk algae blooms pose to growing urban populations. We verified that little literature has been published bridging these knowledge areas.

As it is such a widespread problem and few countries are studying it as an approach of urban environment, which makes it a true Challenge for Public Health, and we recommend further investigations in more countries and hydrographic basins. As mentioned, because these algae are resistant to most water treatment technologies, they pose health risks or they might cause the abandonment of water sources closer to cities, consequently increasing the costs for providing safe water to their inhabitants.

Finally, we call for joint studies and cooperation of laboratories and study groups in limnology, hydrology and environmental health, but also in urban planning, and urban climatology.

## Figures and Tables

**Table 1:** Valid results of mapping literature review on “urban” and “eutrophic” reservoirs.

**Water Quality Studies**					
**Study**	**Country**	**Results**	**Cy**	**OA**	**S/C**
Macrophyte-induced thermal stratification in a shallow urban lake promotes conditions suitable for nitrogen-fixing cyanobacteria. (Vilas et al., 2018) [7]	Australia	Submerged macrophytes can promote blooms of nitrogen-fixing cyanobacteria once they are sufficiently tall and dense to induce stable water column conditions.	y	y	n
Long-term variations in sediment heavy metals of a reservoir with changing trophic states: Implications for the impact of climate change. (Wu et al., 2017) [8]	China	Algae-dominant TOC had strong positive correlation with temperature, which can be explained by increased temperature that accelerated the growth of algae.	n	y	y
Antibiotic resistance genes in surface water of eutrophic urban lakes are related to heavy metals, antibiotics, lake morphology and anthropic impact. (Yang et al., 2017) [9]	China	The results of redundancy analysis and variation partitioning analysis showed that antibiotic and co-selection with heavy metals were the major factors driving the propagation of antibiotic resistance genes in six urban lakes.	n	n	n
Tempo-spatial analysis of water quality in tributary bays of the Three Gorges Reservoir region, China. (Tang et al., 2015) [10]	China	TGR bays had been moderately polluted with averaged nitrogen (N) concentration over 2 mg L^−1^ and phosphorus (P) concentration less than 0.1 mg L^−1^ in dry season, and high P over 0.2 mg L^−1^ and low N of 1.54 mg L^−1^ on average in flooding season. The interaction of dam regulation and flooding events influenced the temporal pattern of water quality in the TGR bays, in which particulate nutrients dynamic played an important role. Urban effluents and agricultural catchment area also influenced water quality in the bays, showing local spatial distribution characteristics via diffusion mechanism.	n	n	y
Nitrous oxide emissions from river network with variable nitrogen loading in Tianjin, China. (Liu et al., 2015) [11]	China	Eutrophied urban rivers in Tianjin were oversaturated with respect to N_2_O with saturation levels ranging from 252% to 3116% and acted as source of N_2_O, whereas rural rivers were generally undersaturated, with saturation levels ranging from 3% to 354% and acted as N_2_O sink.	n	n	y
Seasonal changes of water quality in a tropical shallow and eutrophic reservoir in the metropolitan region of Recife (Pernambuco-Brazil). (De Oliveira et al., 2014) [12]	Brazil	Higher values mean phytoplankton biomass (26.3 mm^3^·L^−1^) occurred in the dry season, especially Planktothrix agardhii and Geiterinema amphibium, which occurred in 100% of samples. High trophic state index was detected throughout the year. Seasonality exerted some influence on both biotic and abiotc variables, leading to changes in water quality of the reservoir.	y	y	y
Responses of periphytic diatoms to mechanical removal of Pistia stratiotes L. in a hypereutrophic subtropical reservoir: dynamics and tolerance. (Matias de Faria et al., 2014) [13]	Brazil	The composition of the diatom community was influenced by seasonal changes in temperature and rainfall. Canonical Correspondence Analyses confirmed a correlation between higher diatom densities and the increased photic zone following macrophyte removal.	n	y	y
Variability of water quality, metals and phytoplankton community structure in urban stormwater ponds along a vegetation gradient. (Vincent et al., 2014) [14]	Canada	The blue-green genus Microcystis was detected in all ponds, and was the dominant taxon in most SPs. This raises concern that SPs may serve as reservoirs of toxin-producing algae. Multivariate analyses of metals, water quality variables, and algal species composition showed considerable dissimilarity among SPs, yet comparably high similarity among reference ponds.	y	y	y
Phytoplankton growth in relation to network topology: time-averaged catchment-scale modeling in a large lowland river. (Istvanovics et al., 2014) [15]	Romania	Growth of river phytoplankton is intrinsically linked with network topology, including spatial distribution of nutrient inputs. Stochastic variability in discharge tends to destabilize, while specific network properties may stabilize phytoplankton growth in large rivers. Shallow rivers support meroplanktonic diatoms that, depending on discharge, may have adequate time to develop summer blooms, making shallow rivers particularly susceptible to eutrophication.	n	y	y
From Eutrophic to Mesotrophic: Modeling Watershed Management Scenarios to Change the Trophic Status of a Reservoir. (Mateus et al., 2014) [16]	Portugal	Model estimates show that a 10% reduction in nutrient loads will suffice to change the state to mesotrophic, and should target primarily Waste Water Treatment Plants effluents, but also act on diffuse sources. The method applied in this study should provide a basis for water environmental management decision-making.	n	n	n
Assessment of organic and inorganic contaminants in sediments of an urban tropical eutrophic reservoir. (Mozeto et al., 2014) [17]	Brazil	The degradation of Ibirité reservoir and its tributaries cannot be solely attributed to the input of hydrocarbons, but predominantly to the discharge of raw urban sewage and effluents from other industrial sources.	n	n	n
Sediment and total phosphorous contributors in Rock River watershed. (Mbonimpa et al., 2014) [18]	USA	Urban land use and agricultural land growing corn rotated with non-leguminous crops are associated with total suspended sediment (TSS) and Total phosphorous (TP) in streams. Increase in corn soybean rotation acreage within the watershed is associated with reduction in stream’s TSS and TP. Forest and water bodies were associated with reduction in TSS and TP. The authors recommend adoption of the Low Impact Development (LID) approach in urban dominated subwatersheds.	n	n	n
Assessment of Cyanobacterial toxicogenic genotypes and estimation of toxin in urban lakes. (Morais et al., 2014) [19]	Portugal	The presence of toxic cyanobacteria and their toxins was detected throughout the sampling period, from July 2008 until February 2009. The highest amount of microcystins (MC) reached 10.2 µg MC-LR equivalents/L in November.	y	n	y
Past, Present, and Future Nutrient Quality of a Small Southeastern River: A Pre-Dam Assessment. (Miller et al., 2013) [20]	USA	Historical and current nutrient concentrations were elevated throughout the watershed, in most cases above suggested criteria, and indicated that water quality of the river was and continues to be nutrient rich. A future reservoir at recent levels of water quality will likely be highly eutrophic, and anthropogenic influences will further stress this ecosystem and its water quality as the urban region expands.	n	n	y
Understanding the patterns and mechanisms of urban water ecosystem degradation: phytoplankton community structure and water quality in the Qinhuai River, Nanjing City, China. (Zhao et al., 2013) [21]	China	Canonical correspondence analysis (CCA) between environmental parameters and phytoplankton communities showed that Chlorophyta could tolerate the higher concentrations of the permanganate index, nitrogen, and phosphorus in eutrophic water; Bacillariophyta could adapt well to changing water environments; and the TN/TP ratio had obvious impacts on the distributions of Cyanophyta, Euglenophyta, and some species of Chlorophyta. CCA analyses for autumn and winter data revealed that the main environmental parameters influencing phytoplankton distribution were water temperature, conductivity, and total nitrogen.	y	y	y
The influence of land use and occupation on the quality and genotoxicity of water in the Itupararanga reservoir, Sao Paulo, Brazil. (Taniwaki et al., 2013) [22]	Brazil	The quality of water and sediment at the Itupararanga reservoir is compromised by land use, human occupation and natural vegetation removal, requiring emergency managements to ensure the sustainability of water resources.	n	y	n
Environmental quality of the La Polvora urban lagoon in the Grijalva river watershed. (Sanchez et al., 2012) [23]	Mexico	This lagoon is undergoing environmental degradation, as indicated by its hyper-eutrophic state, high values for fecal coliforms, low values for diversity of epibenthic macrofauna and fish, the percentage of exotic species and low values for the conditional factor of 11 fish species. Findings suggest the need to review the management program and its operations in this ecosystem.	n	n	y
Algae community and trophic state of subtropical reservoirs in southeast Fujian, China. (Yang et al., 2012) [24]	China	The degradation of water quality associated with excess levels of nitrogen and phosphorus in Fujian reservoirs may be impacted by interactions among agriculture and urban factors. A watershed-based management strategy, especially phosphorus control, should be developed for drinking water source protection and sustainable reservoirs.	y	y	y
Response of cyanobacteria and algae community from small water bodies to physicochemical parameters. (Richter et al., 2012) [25]	Poland	Results distinguished four groups of basins (I—artificial basins within urban areas; II—old river-beds within urban areas; III—ponds in rural areas; IV—an old river-bed in forest areas). Distinction shows major relevance of reservoirs’ origins and their presence in the landscape. Additional principal components analysis (PCA) and redundancy analysis (RDA) of the basins have shown that the biological parameters are more efficient in diversifying the basins in respect of their origins than the physicochemical parameters.	y	y	n
Hydrochemical Consequences of Feeding Flow-through Reservoirs with Contaminated Water. (Jagus et al., 2012) [26]	Poland	Individuality of reservoirs in terms of their impact on fluvial transportation of substances. Each reservoir fed with contaminated water will have at least partly (selectively) contaminating impact in the course of time. That should be related to increasing eutrophication in conditions of limited capacity of accumulation of contaminants in the limnic environment.	n	n	n
Effects of air-drying on phosphorous sorption in shallow lake sediment, China. (Xiao et al., 2012) [27]	China	In contrast with the field-moist samples, the air-dried ones showed a significantly positive relationship between organic matter (OM) content and EPC0 value, suggesting that the breakdown of OM during drying may be a major source of P upon reflooding.	n	n	n
Limiting factors for phytoplankton growth in subtropical reservoirs: the effect of light and nutrient availability in different longitudinal compartments. (Fernandes et al., 2011) [28]	Brazil	Putting Itupararanga reservoir in a regional context allowed assessment of potential influences of land use on trophic state. Within the subtropical dataset, TP explained a greater percentage of variance in Chl-a (*R*^2^ = 0.70) than T’N (*R*^2^ = 0.17). The main land use type within the reservoirs drainage area significantly influenced the concentrations of TP, TN, and Chl-a (*p* 0.05, MANOVA), with different relationships between nutrients and chlorophyll in forested (*R*^2^ = 0.12–0.33), agricultural (*R*^2^ = 0.50–0.68) and urban (*R*^2^ = 0.09–0.64) watersheds	n	y	y
Phytoplankton as a monitoring tool in a tropical urban shallow reservoir (Garças Pond): the assemblage index application. (Crossetti et al., 2008) [29]	Brazil	Twenty one functional groups ‘sensu’ Reynolds were identified. Cyanobacteria contribution played the main role during the drastic alterations that occurred after water hyacinth removal. Results of ecological status of reservoir using Q index showed statistical difference among the 3 limnological phases (one way ANOVA; F = 119.4; *p* = 0.000). Regarding Q index classification, Garças Reservoir limnological phases were characterized as follows: (1) phase I: 0 ≥ Q ≤ 2.9, medium to bad; (2) phase II: 1.4 ≥ Q ≤ 3, tolerable to medium; and (3) phase III: 0 ≥ Q ≤ 1.5, bad to tolerable ecological states.	y	y	n
The importance of excretion by Chironomus larvae on the internal loads of nitrogen and phosphorus in a small eutrophic urban reservoir. (Henry et al., 2008) [30]	Brazil	Dependence in relation to recorded temperature for the ammonium and phosphate excretions was significantly higher at 25 °C than at 20 and 15 °C. Values showed that internal loads by excretion from Chironomus larvae correspond to approximately 33% of the external loads of phosphorus in the lake and, in the case of nitrogen, to only 5%.	y	y	n
Enhanced source-water monitoring for New York City: summary and perspective. (Sweeney et al., 2006) [31]	USA	Streams and rivers located west of Hudson River (WOH) deliver good to very good water to most of the receiving reservoirs. The project confirmed the eutrophic condition of the WOH Cannonsville reservoir and further linked that condition to nutrient inputs from the West Branch Delaware River. The project also confirmed that many streams located east of Hudson River (EOH) had fair to poor water quality and that streams in the Croton and Kensico watersheds were biologically and functionally degraded. Anthropogenic changes in land use from forested to agricultural in the WOH region have affected water chemistry, macroinvertebrate community structure, and stream function, although the impact is less than that caused by changes in land use from forested to urban in the EOH region.	n	n	n
Use of robotic monitoring to assess turbidity patterns in Onondaga Lake, NY. (Effler et al., 2006) [32]	USA	Major runoff events are demonstrated to cause conspicuous short-term increases in Tn that are manifested as metalimnetic peaks in summer and early fall, associated with the entry of the negatively buoyant primary tributary source as an interflow. Annual occurrence of Tn maxima within the oxycline of the metalimnion in October.	n	n	y
Phytoplankton characteristics, trophic evolution, and nutrient dynamics in an urban eutrophic lake: Kandy Lake in Sri Lanka. (Silva, 2005) [33]	Sri Lanka	Pediastrum simplex, a Chlorophyte commonly found in eutrophic tropical waters, became virtually absent following the outbreak of M. aeruginosa bloom, while Aulacoseira granulata, a chain forming centric diatom, a climax species in the tropics, declined dramatically during post-blooming. The outbreak of cyanobacteria changed the functional phytoplankton groups from a rhythmically oscillating Chlorophyte and a Diatomophyceae to a multiple assemblage of Cyanophytes. The upper level of the annual mean chlorophyll-a, which showed an exponential increase over a period of seven years, had a negative correlation with the lower limit of the annual mean water transparency.	y	y	y
Limnological and loading information and a phosphorus total maximum daily load (TMDL) analysis for Onondaga Lake. (Effler et al., 2002) [34]	USA	The phosphorus (P) total maximum daily load (TMDL) analysis is demonstrated to understate the present role of the dominant point source and overstate the importance of non-point sources. Recommendations are made to upgrade the TMDL analysis through an integrated program of model development, testing and application, supporting process studies and monitoring, and re-evaluation of management options.	n	y	y
Nutrient budget for Saguling Reservoir, West Java, Indonesia. (Hart et al., 2002) [35]	Indonesia	Saguling Reservoir receives a very large nutrient load from the city of Bandung becoming highly eutrophic. It is unlikely that water quality of Saguling will improve until substantial part of Bandung is sewered and adequate discharge controls are placed on the many industries in the region upstream of the reservoir.	n	n	n
Water quality in drinking water reservoirs of a megacity, Istanbul. (Baykal et al., 2000) [36]	Turkey	Significance of site selection for urban activities, construction, settlements, and industry to sustain acceptable water quality in the drinking water reservoirs, which in turn will provide a higher quality of urban life for the inhabitants of the megacity.	n	n	n
Phytoplankton community composition in relation to water quality and water-body morphometry in urban lakes, reservoirs, and ponds. (Olding et al., 2000) [37]	Canada	Nutrient control is the primary issue, and one of the reservoirs has already exceeded the limits of being eutrophic, one is at mesotrophic conditions, and the remaining four are at the limit of being eutrophic, indicating the significance of making the correct decision and taking pertinent measures for management and control. The only mesotrophic resource has no industry and a very low population density, whereas the one that is already eutrophic has the highest population density, and the greatest percentage of urban land use within its watershed.	y	y	y
Eutrophication gradients in coastal lagoons as exemplified by the Bassin d’Arcachon and the Etang du Prevost. (Castel et al., 1996) [38]	France	Three stages of eutrophication are recognized according to the conceptual model of Nienhuis describing the relation between the relative dominance of primary producers connected to the availability of nutrients. Such macroscopic observations should, now, be explained by the study of microbiological processes.	n	y	y
**Limnological Life Dynamics Studies**					
**Study**	**Country**	**Results**	**Cy**	**OA**	**S/C**
Diet of an invading clupeid along an urban neotropical reservoir: responses to different environmental conditions. (Nanini-Costa et al., 2017) [39]	Brazil	Considering spatial differences in the consumed zooplankton, the analysis of percentage similarity (SIMPER) indicated the highest dissimilarity (95.3%) between the Boror, (hypereutrophic) and Grande River Reservoir (supereutrophic) sites, while trophic niche width increased from the most impacted (Boror) to the most preserved site (Capivari).	n	n	n
Cyanobacteria bloom: selective filter for zooplankton? (Mello et al., 2015) [40]	Brazil	Cyanobacteria blooms at Ibirité reservoir are acting as “selective filters”, and are, thus, disturbances with sufficient ability to change the structure of the zooplankton community.	y	n	y
Does the plankton community follow the horizontal water quality heterogeneity in a tropical urban reservoir (Guarapiranga reservoir, São Paulo, Brazil)? (Nishimura et al., 2014) [41]	Brazil	Different outcomes indicate that the water quality, phytoplankton and zooplankton communities captured different features from the epilimnetic layer in the longitudinal axis of the Guarapiranga reservoir. Importantly, the phytoplankton and zooplankton communities in the dam region were altered, directly or indirectly, by copper sulphate treatment.	n	y	n
Effects of trophic levels (chlorophyll and phosphorous content) in three different water bodies (urban lake, reservoir and aquaculture facility) on gill morphology of Nile tilapia (Oreochromis niloticus). (Shimada Borges et al., 2013) [42]	Brazil	A significant difference in the histological alterations index (HAI) was observed only in fish from the urban lake, with the presence of cell hypertrophy, hyperplasia, aneurism, and other alterations. When compared to the other groups, a large quantity of rodlet cells was also observed in the urban group. These results demonstrate the correlation of eutrophic states of water with gill morphology. Also discussed is the premise that large amounts of organic material dissolved in water can alter the morphology of the fish gills.	n	y	n
Do phytoplankton fractions < 20 μm dominate in tropical reservoirs independent of their trophy? (Gil-Gill, 2011) [43]	Brazil	Both total and fractioned production were distinct during rain and dry periods and stratification and mixing processes. Light was the limiting factor.	n	y	y
Diversity of larvae of littoral Chironomidae (Diptera: Insecta) and their role as bioindicators in urban reservoirs of different trophic levels. (Morais et al., 2010) [44]	Brazil	Significant differences in the taxonomic composition, richness, equitability, and diversity of the chironomid assemblages in these three reservoirs of different trophic levels.	n	n	y
Seasonal Dynamics of Phytoplankton in Relation to Key Aquatic Habitat Factors in a Polluted Urban Small Water Body in Tianjin, China. (Sun et al., 2009) [45]	China	The dominant cyanobacterial species Oscillatoria tenuis Ag. bloomed in autumn, other species with higher degrees of dominance never bloomed in the year investigated. Significant correlations between habitat factors and phytoplankton biomass.	y	y	y
Adaptations in phytoplankton life strategies to imposed change in a shallow urban tropical eutrophicreservoir, Garças Reservoir, over 8 years. (Crossetti et al., 2008) [46]	Brazil	The growth of Eichhornia crassipes biomass in response to increase in allochthonous input of N and P to Garças Reservoir promoted limnological alterations in three distinct phases. Changes in the structure and dynamics of the phytoplankton community directly associated with its survival strategies in those three phases.	y	y	y
Temporal fluctuation and reproduction of Thermocyclops decipiens (Copepoda, Cyclopoida) in an eutrophic lake of central Brazil. (Padovesi-Fonseca et al., 2002) [47]	Brazil	Ecological factors that determine the success of T. decipiens in eutrophic systems are related to omnivorous feeding habits and prey-predator interactions	n	n	y
Diatom succession in an urban reservoir system. (Donar et al., 1995) [48]	USA	Prior to the establishment of the reservoir, the diatom flora was dominated by benthic taxa. Benthic diatoms were numerous throughout the entire core, but eutrophic taxa dominated much of the core after the reservoir’s creation. Total diatom density increased about tenfold in the about the first 10–15 years after the reservoir’s creation before declining markedly.	n	n	n
The abundance of epizoic ciliate epistyli-daphniae related to their host moina-macrocopa in na urban stream. (Xu, 1992) [49]	China	In midsummer, mean number of epizoites per host reached its peak (more than 80) associated with lower abundance of M. macrocopa. Other relationships between number of Epistylis daphniae zooids per M. macrocopa and various aspects of the M. macrocopa life cycle were studied.	n	n	y
**Restoration Studies**					
**Study**	**Country**	**Results**	**Cy**	**OA**	**S/C**
Changes in Phytoplankton and Water Quality during Sustainable Restoration of an Urban Lake Used for Recreation and Water Supply. (Kozak et al., 2017) [50]	Poland	Comparison of data with pre-restoration data showed that, as a result of treatments orthophosphates decreased, rarely exceeding 0.06 mg P·L^−1^, and cyanobacterial water blooms disappeared. Deep chlorophyll maximum occurred in summer at a depth of 5 m as result of restoration, confirming lower trophic status of the lake, alluding to mesotrophic conditions. Cyanobacteria were found in the lake but they were not abundant	y	y	y
The efficiency of combined coagulant and ballast to remove harmful cyanobacterial blooms in a tropical shallow system. (Miranda et al., 2017) [51]	Brazil	Polyaluminium chloride combined with ballast was effective in settling blooms dominated by Microcystis or Cylindrospermopsis. Chitosan combined with ballast was only effective in settling Cylindrospermopsis-dominated blooms at low pH, whereas at pH ≥ 8 no effective flocculation and settling could be evoked. Chitosan also had a detrimental effect on Cylindrospermopsis causing the release of saxitoxins.In contrast, no detrimental effect on Microcystis was observed and all coagulant-ballast treatments were effective in settling the Microcystis dominated bloom, and in lowering dissolved microcystin concentrations. The best procedure for biomass reduction also depends on the dominant species.	y	y	n
Algal blooms, circulators, waterfowl, and eutrophic Greenfield Lake, North Carolina. (Mallin et al., 2016) [52]	USA	A year-long survey indicated that waterfowl, especially cormorants, contributed somewhat to the lake’s total N load but a considerable amount of P, particularly in winter.The lake sediments apparently function as P reservoirs, supplying P to the water column during summer, setting the stage for runoff-induced nitrate pulses and subsequent algal blooms. The increased chlorophyll a violations have led to inclusion of the lake on the North Carolina 303 (d) list of impaired waters.	y	y	y
Evaluation of the suitability of anthropogenic reservoirs in urban space for ecological restoration using submerged plants (Upper Silesia, Poland). (Pierzchala et al., 2016) [53]	Poland	Reservoirs are different in the evaluated potential (P: from −1 to 3) and suitability (S: from −3 to −1). The 3 proposed stages of evaluation of reservoirs’ potential and suitability to be adapted to enable the categorization and determination of bodies of water for restoration activities. At present, mine subsidence reservoirs constitute the majority of anthropogenic reservoirs in the post-mining areas.	n	y	y
Ecotoxicological risks of calcium nitrate exposure to freshwater tropical organisms: Laboratory and field experiments. (Sueitt et al., 2015) [54]	Brazil	Laboratory and field data to assess the ecotoxicological risks of calcium nitrate exposure to freshwater tropical biota.	y	y	n
Influence of different recultivation methods on durability of nitrogen compounds changes in the waters of an urban lake. (Grochowska et al., 2015) [55]	Poland	Increase of nitrate nitrogen concentration to about 1.30 mg/L. A tenfold decrease in ammonia and TN concentration as result of the artificial aeration. Concentrations of nitrogen compounds noted 8 years after termination of the lake recultivation were typical for reservoirs with moderate trophic status.	n	n	n
The influence of different recultivation methods on the water buffer capacity in a degraded urban lake. (Grochowska et al., 2013) [56]	Poland	Observed changes after restoration of Dlugie Lake are very positive in that they are optimal for construction of plant cell walls, shells and fish bones.	n	n	n
Calcium nitrate addition to control the internal load of phosphorus from sediments of a tropical eutrophic reservoir: Microcosm experiments (Yamada et al., 2012) [57]	Brazil	Calcium nitrate treatment proved to be a valuable tool in remediation of eutrophic aquatic ecosystems leading to conditions that can support a great diversity of organisms after a restoration period.	n	n	n
Assessment of the acute toxicity of eutrophic sediments after the addition of calcium nitrate (Ibirite reservoir, Minas Gerais-SE Brazil): initial laboratory experiments. (Janke et al., 2011) [58]	Brazil	Method was inefficient for restoration of sediments with nitrate addiction.	n	n	n
Structural and functional phytoplankton responses to nutrient impoverishment in mesocosms placed in a shallow eutrophic reservoir (Garças Pond), São Paulo, Brazil. (Crossetti et al., 2005) [59]	Brazil	The initial community mainly represented by R- and S-strategists (Planktothrix, Cylindrospermopsis and Microcystis) were gradually replaced by C-strategists (Cryptomonas spp., Chloroccoccales in general). Characteristics of the initial succession phases were observed in all treatments. The community was first inhabited by fast growing species but no important biomass contribution of size fractions was observed. Space liberated by blue-green species that did not adapt to the new nutrient-impoverished conditions was gradually occupied by other algal species, which together contributed to most of the total biomass registered in all treatments.	y	y	n

y = yes; n = no; Cy = Cyanobacteria; OA = Other Algeae; C/S = climate/seasonality. Country = where reservoir is located. TOC: Total Organic Carbon; SPs: Stormwater Ponds; TGR: Three Gorges Reservoir; TN: Total Nitrogen; TP: Total Phosphorus; MANOVA: Multivariate Analysis of Variance; ANOVA: Analysis of Variance. Water Quality Studies: studies that aimed monitor the water quality of reservoirs. Limnological Life Dynamics Studies: the ones that aimed at the dynamics and peculiarities of living organisms in the limnological/aquatic environment. Restoration Studies: studies that reported cases and techniques for reservoir recovery.
